# Anti-Skin Aging Potential of Methoxyflavones from *Kaempferia parviflora* Against TNF-α-Induced Oxidative Stress and Photoaging in Normal Human Dermal Fibroblasts

**DOI:** 10.3390/foods14234012

**Published:** 2025-11-23

**Authors:** Si-young Ahn, Se Yun Jeong, Bum Soo Lee, Yun Seok Joh, Hamed Hamishehkar, Sullim Lee, Ki Hyun Kim

**Affiliations:** 1 Department of Life Science, College of Bio-Nano Technology, Gachon University, Seongnam 13120, Republic of Korea; sy990303@gachon.ac.kr; 2 School of Pharmacy, Sungkyunkwan University, Suwon 16419, Republic of Korea; dlawktkark@naver.com (S.Y.J.); kosboybs@naver.com (B.S.L.); ysjoh05@g.skku.edu (Y.S.J.); 3 Drug Applied Research Center, Tabriz University of Medical Sciences, Tabriz 51368, Iran; hamishehkarh@tbzmed.ac.ir; 4 Research Center of New Material and Green Chemistry, Khazar University, 41 Mehseti Street, Baku AZ1096, Azerbaijan

**Keywords:** *Kaempferia parviflora*, methoxyflavones, anti-skin aging, TNF-α, normal human dermal fibroblasts

## Abstract

Reactive oxygen species (ROS) generated by ultraviolet (UV) radiation accelerate skin aging by activating matrix metalloproteinase-1 (MMP-1) and mitogen-activated protein kinase (MAPK) signaling pathways. Therefore, antioxidants that can suppress ROS generation and downstream signaling cascades are considered promising anti-aging agents. In this study, five methoxyflavones were isolated from *Kaempferia parviflora* (black ginger) rhizomes—5,7,3′,4′-tetramethoxyflavone (**1**), 3,5,7,4′-tetramethoxyflavone (**2**), 5,7,4′-trimethoxyflavone (**3**), 3,5,7,3′,4′-pentamethoxyflavone (**4**), and 5,7-dimethoxyflavone (**5**)—using LC–MS-guided fractionation and identified via NMR and LC–MS analysis. Their biological activities were evaluated in tumor necrosis factor-α (TNF-α)-stimulated normal human dermal fibroblasts (NHDFs). All methoxyflavones, except compound **3**, significantly suppressed TNF-α-induced ROS generation, while compounds **3**–**5** markedly reduced MMP-1 secretion. Among them, compounds **4** and **5** exerted the strongest protective effects by modulating distinct MAPK pathways: compound **4** selectively inhibited p38 phosphorylation, whereas compound **5** selectively suppressed ERK phosphorylation. Both compounds attenuated ECM degradation and enhanced antioxidant defenses in a concentration-dependent manner. Collectively, these findings highlight the mechanistic significance of methoxyflavones **4** and **5** as dual-acting antioxidant and ECM-protective agents that counteract skin aging through selective regulation of MAPK signaling. Their potential as natural anti-photoaging ingredients warrants further validation in in vivo models and clinical studies for future skincare applications.

## 1. Introduction

The skin functions as a critical protective barrier that shields the body from environmental insults and consists of multiple layers [1]. The epidermis, which forms the outermost part of the skin, is primarily made up of keratinocytes, while the dermis beneath it contains abundant collagen and elastin fibers, contributing to the skin’s resilience and tensile strength. Below the dermis lies the subcutaneous adipose tissue, which cushions the body and helps regulate temperature [2]. However, as individuals age, the skin undergoes various structural and functional deteriorations. These aging processes can be classified into two main types: intrinsic aging and extrinsic aging [3]. Intrinsic aging, which is genetically driven, involves a reduction in collagen synthesis, degradation of elastin networks, and a diminished capacity to retain moisture [4,5]. In contrast, extrinsic aging, also known as photoaging, is predominantly triggered by environmental exposures—most notably ultraviolet (UV) radiation [6,7]. UV light penetrates deep into the skin and stimulates tumor necrosis factor-alpha (TNF-α) receptors, initiating the formation of reactive oxygen species (ROS), which cause extensive cellular and extracellular matrix damage [8,9]. ROS are key contributors to cutaneous aging, as they oxidize essential biomolecules including DNA, proteins, and lipids, thereby compromising the skin’s structural and functional integrity [10,11]. Importantly, ROS also enhance the activity of matrix metalloproteinase-1 (MMP-1), an enzyme that breaks down collagen fibers, resulting in decreased skin firmness and the appearance of wrinkles [12]. Moreover, ROS activate the mitogen-activated protein kinase (MAPK) signaling pathway, which in turn upregulates the activator protein-1 (AP-1) transcription factor, further promoting MMP-1 expression [13]. The MAPK cascade plays a pivotal role in modulating cellular responses relevant to skin aging, and AP-1 intensifies collagen breakdown through transcriptional activation of MMP-1 [14,15]. Consequently, ROS produced by UV exposure are central mediators of photoaging, facilitating dermal degradation via MMP-1 and MAPK/AP-1 pathways [16,17,18]. Elucidating these molecular mechanisms offers essential insights into the aging process of the skin and highlights potential therapeutic targets for the development of anti-aging interventions.

Maintaining skin health and appearance is widely regarded as a reflection of overall wellness. In recent years, interest in skin rejuvenation methods has surged, with global expenditures on such treatments projected to grow from $24.6 billion to around $44.5 billion [19]. A variety of methods—such as energy-based devices, chemical exfoliants, and injectable treatments—are currently employed to enhance skin condition [19]. Among these, topical skincare remains the most accessible and non-invasive approach, making it a popular choice for individuals pursuing anti-aging benefits [19]. At the same time, growing public concern over the adverse effects of synthetic chemicals found in traditional cosmetic products has accelerated the expansion of the natural skincare market [20]. This shift in consumer preference is supported by innovations in cosmetic science and an increasing demand for safer yet efficacious alternatives [20]. Natural compounds are especially valued for their safety profile and therapeutic potential, offering a wide range of pharmacological activities, including antioxidant, anti-inflammatory, anti-aging, and anticancer effects [21]. Furthermore, their natural abundance and renewability make them appealing from a sustainability standpoint [20]. Numerous studies have highlighted the anti-aging efficacy of phytochemicals—such as flavonoids, polyphenols, quinones, monoterpenes, and polysaccharides—isolated from diverse natural sources in preclinical models [21]. In line with this evidence, our study investigates the protective effects of naturally derived bioactive molecules against TNF-α-induced damage in human dermal fibroblasts (HDFs) [22]. This approach utilizes the chemical richness of natural products to drive innovation in therapeutic agent development.

The genus *Kaempferia*, belonging to the family Zingiberaceae, comprises a notable group of medicinal plants found predominantly in India, Malaysia, South China, and Thailand. Among its various members, *K. galanga*, *K. rotunda*, and *K. parviflora* are the most widely recognized species [23,24]. Notably, *Kaempferia parviflora* Wall. has gained attention as a functional ingredient in health foods and dietary supplements, attributed to its promising effects on general health and physical performance enhancement [25]. Traditionally, the rhizomes of *K. parviflora* have been used to manage digestive issues, fungal diseases, and allergic reactions. Scientifically, it has been reported to possess a wide spectrum of pharmacological properties, including anti-inflammatory, antiallergic, antifungal, antispasmodic, antiplasmodial, antimycobacterial, and anti-HIV-1 activities [26]. Phytochemical investigations have revealed that *K. parviflora* contains various bioactive constituents such as phenolic glycosides, sesquiterpenes, flavones, flavanones, diarylheptanoids, and chalcones [27,28,29]. Among these, a sesquiterpene of the cadinane type has demonstrated potent immunotoxicity against the early fourth-instar larvae of *Aedes aegypti* mosquitoes [28]. Chalcones such as flavokavins A and B, as well as curcumin derivatives, have been identified as strong suppressors of nitric oxide (NO) production, while flavanones and stilbenes from this plant showed comparatively weaker effects [29]. Methoxyflavones, the major constituents of *K. parviflora*, have been reported to exhibit multiple pharmacological benefits, including anti-inflammatory, anticancer, antioxidant, neuroprotective, cardioprotective, and antiallergic properties [30]. Notably, 5,7,4′-trimethoxyflavone and 5,7-dimethoxyflavone have demonstrated potent inhibitory effects on acetylcholinesterase (AChE) and butyrylcholinesterase (BChE), suggesting their therapeutic potential in treating Alzheimer’s disease [31]. Additionally, polymethoxyflavones (PMFs) derived from *K. parviflora* have been shown to inhibit degranulation in RBL-2H3 mast cells, indicating their role in alleviating type I hypersensitivity reactions [32]. Extracts containing 5,7-dimethoxyflavone and 3,7,3′,4′-tetramethoxyflavone have also demonstrated marked anti-inflammatory and antioxidant capacities in vitro, suggesting their utility in preventing atherosclerosis progression [33].

Although polymethoxyflavones (PMFs) exhibit strong antioxidant and anti-inflammatory activities, their roles as anti-aging agents in impaired human dermal fibroblasts (HDFs) have only been explored for a limited number of specific compounds. For instance, several PMFs—namely 5,7-dimethoxyflavone, 5,7,4′-trimethoxyflavone, and 3,5,7,3′,4′-pentamethoxyflavone—isolated from *Kaempferia parviflora* have been shown to attenuate cellular senescence, reduce intracellular ROS levels, and suppress the senescence-associated secretory phenotype (SASP) in primary HDFs [34]. In our recent investigations using TNF-α-stimulated normal human dermal fibroblasts (NHDFs), both 5,7,4′-trimethoxyflavone and 3,5,7-trimethoxyflavone emerged as highly bioactive methoxyflavones. These compounds not only significantly downregulated MMP-1 expression but also upregulated collagen type I and COLIA1, inhibited ROS generation induced by TNF-α, and reduced the expression of pro-inflammatory markers such as COX-2, IL-1β, and IL-6. Simultaneously, they enhanced heme oxygenase-1 (HO-1) levels and interfered with the MAPK signaling cascade by downregulating the activity of AP-1 and NF-κB, both of which play key roles in dermal fibroblast inflammation and degradation [35,36]. Given their promising anti-aging potential and the relatively limited scope of current research, there is a strong rationale for conducting further studies to identify and characterize novel methoxyflavones from *K. parviflora* with skin-protective properties.

As part of our continuous exploration of bioactive constituents from diverse natural origins [37,38], we extended our investigation to identify anti-skin aging methoxyflavones from *K. parviflora*. Utilizing a combination of repeated column chromatography and high-performance liquid chromatography (HPLC), followed by structural confirmation through liquid chromatography–mass spectrometry (LC–MS), we successfully isolated five distinct methoxyflavones (compounds **1**–**5**) from the methanol (MeOH) extract of *K. parviflora* rhizomes. Considering the encouraging findings from earlier studies on the anti-aging efficacy of selected polymethoxyflavones (PMFs) in stressed human dermal fibroblasts (HDFs), this study sought to assess whether the isolated methoxyflavones could counteract TNF-α-mediated inflammatory damage associated with skin aging. Herein, we present the isolation and structural elucidation of methoxyflavones 1–5 and evaluate their protective effects against TNF-α-induced aging phenotypes in normal human dermal fibroblasts (NHDFs), with an emphasis on analyzing their structure–activity relationships (SARs).

## 2. Materials and Methods

### 2.1. Plant Material

Rhizomes of *K. parviflora* were collected in January 2020 from Warorot Market, located in Chiang Mai City, Northern Thailand, and the detailed information of plant material is included in Supplementary Materials.

### 2.2. Extraction and Isolation of Compounds

A total of 132.0 g of dried *Kaempferia parviflora* rhizomes was finely cut and extracted three times with 80% aqueous methanol (MeOH). The pooled methanolic extracts were concentrated under reduced pressure to obtain a crude extract weighing 9.2 g. This crude material was then suspended in 700 mL of distilled water and subjected to successive liquid–liquid partitioning with solvents of increasing polarity: *n*-hexane (Hx), dichloromethane (CH_2_Cl_2_), ethyl acetate (EtOAc), and *n*-butanol (*n*-BuOH). As a result, four distinct solvent fractions were obtained: an *n*-hexane-soluble fraction (1.0 g), a CH_2_Cl_2_-soluble fraction (3.2 g), an EtOAc-soluble fraction (0.4 g), and an *n*-BuOH-soluble fraction (0.5 g). The CH_2_Cl_2_ fraction (3.2 g), which yielded the largest mass among the fractions, was further purified by normal-phase silica gel column chromatography using a gradient elution of *n*-hexane and ethyl acetate (from 10:1 to 0:100, *v*/*v*), resulting in five subfractions designated as A through E. Fraction C (2.3 g) was further purified by reversed-phase C18-silica gel column chromatography with a gradient solvent system (MeOH/H_2_O, 80:20 → 100:0), yielding three fractions (C1–C3). Fraction C2 (2.2 g) was then subjected to Sephadex LH-20 column chromatography using methanol as the mobile phase, resulting in two fractions (C21–C22). Subsequently, fraction C21 (2.0 g) underwent silica gel column chromatography with a gradient solvent system (CH_2_Cl_2_/MeOH, 50:1 → 0:100), yielding six subfractions (C211–C216). Fraction C211 (1.2 g) was further separated by preparative HPLC using a gradient solvent system (acetonitrile (MeCN)/H_2_O, 55:45 → 100:0) to yield four fractions (C2111–C114). Fraction C2112 (70 mg) was purified by semi-preparative HPLC employing an isocratic solvent system (MeOH/H_2_O, 75:25), resulting in the isolation of compounds **1** (*t*_R_ = 25.0 min, 1.2 mg), **2** (*t*_R_ = 37.0 min, 4.6 mg), and **3** (*t*_R_ = 32.5 min, 7.4 mg). Similarly, fraction C2113 (72 mg) was purified by semi-preparative HPLC using an isocratic solvent system (MeCN/H_2_O, 50:50), leading to the isolation of compounds **4** (*t*_R_ = 16.7 min, 17.3 mg) and **5** (*t*_R_ = 19.8 min, 11.0 mg).

### 2.3. Cell Culture and Sample Preparation

Normal human dermal fibroblasts (NHDFs) were sourced from PromoCell GmbH (Heidelberg, Germany), and the detailed information of cell culture and sample preparation is included in Supplementary Materials.

### 2.4. Cell Viability Measurement

The detailed information for cell viability of the isolated compounds on NHDFs is included in Supplementary Materials.

### 2.5. Measurement of Intracellular ROS Activity

To evaluate the intracellular reactive oxygen species (ROS) levels induced by the isolated compounds, NHDFs were seeded into 96-well plates at a density of 1 × 10^4^ cells per well and incubated for 24 h. To synchronize the cell cycle, cells were subsequently cultured in serum-free DMEM for another 24 h. One hour following treatment with the test compounds, the control group was treated with 20 ng/mL of 2′,7′-dichlorofluorescein diacetate (DCFDA), while the experimental groups were co-treated with both DCFDA and TNF-α at concentrations of 20 ng/mL each. A 15-min TNF-α stimulation period was used to quantify the rapid and transient intracellular accumulation of reactive oxygen species (ROS), which typically occurs during the early phase of oxidative stress induction. After a 15-min incubation period, fluorescence intensity was quantified at 485/535 nm using a microplate reader. For visualization of ROS accumulation, NHDFs were seeded into 48-well plates at a density of 2 × 10^4^ cells per well and cultured for 24 h under identical conditions. The same treatment procedure was followed, and DCFDA-stained cells were immediately examined under a fluorescence microscope (Olympus, Tokyo, Japan).

### 2.6. Enzyme-Linked Immunosorbent Assay (ELISA)

To measure the expression levels of MMP-1 and Pro-Collagen I α1 (COLIA1) proteins, NHDFs were seeded into 96-well plates at a density of 2 × 10^4^ cells per well and incubated for 24 h. To synchronize cell growth, the medium was replaced with serum-free DMEM, and the cells were cultured for an additional 24 h. Subsequently, the cells were pretreated with the test samples for 1 h, followed by stimulation with TNF-α at a final concentration of 20 ng/mL. A 24-h TNF-α stimulation period was used to ensure sufficient time for the transcription, translation, and extracellular secretion of MMP-1, allowing for the accurate quantification of the secreted protein in the supernatant. After 24 h of incubation, the supernatants were collected for protein quantification. Levels of MMP-1 and COLIA1 were determined using the Human Total MMP-1 DuoSet ELISA kit and Human Pro-Collagen I α1 DuoSet ELISA kit (R&D Systems, Minneapolis, MN, USA), and absorbance was recorded at 450 nm with a microplate reader.

### 2.7. Western Blotting

NHDFs were seeded into 6-well culture plates at a density of 3 × 10^5^ cells per well and incubated for 24 h. To synchronize the cells, the culture medium was replaced with serum-free DMEM, and the cells were maintained under these conditions for an additional 24 h. After synchronization, cells were treated with compounds **4** and **5** for 1 h, followed by stimulation with TNF-α at a concentration of 20 ng/mL. A 15-min TNF-α treatment period was selected to capture the rapid and maximal phosphorylation of ERK and p38 proteins, representing key early events in the TNF-α-induced signaling cascade. Fifteen minutes after TNF-α treatment, cells were collected for the analysis of phosphorylated and total forms of ERK and p38, along with GAPDH as a loading control. Cell lysates were prepared using 1× RIPA buffer (Tech & Innovation, Gangwon, Republic of Korea), and protein concentrations were determined using a BCA protein assay kit (Merck, Darmstadt, Germany). Equal amounts of protein were resolved via SDS-PAGE and subjected to Western blot analysis using specific primary antibodies targeting phospho-ERK, ERK, phospho-p38, p38, and GAPDH (Cell Signaling Technology, Danvers, MA, USA). Membranes were incubated with primary antibodies for 4 h at 20 ± 5 °C. After incubation with the primary antibodies, the membranes were treated with HRP-linked goat anti-rabbit IgG secondary antibodies (Cell Signaling Technology) for 1 h at room temperature. The immunoreactive signals were visualized using the SuperSignal™ West Femto Maximum Sensitivity Chemiluminescent Substrate (Thermo Fisher Scientific, Waltham, MA, USA) and captured with a Fusion Solo imaging system (PEQLAB Biotechnologie GmbH, Erlangen, Germany). The band densities were normalized to GAPDH and expressed as percentages relative to the untreated control samples.

### 2.8. Statistical Analysis

All experimental results are expressed as the mean ± standard error of the mean (SEM). Statistical analyses were conducted using GraphPad Prism software (version 9) with one-way ANOVA, followed by Tukey’s multiple comparison test. Differences were considered statistically significant when the *p*-value was less than 0.05.

## 3. Results

### 3.1. Isolation and Structural Identification of Methoxyflavones

**Figure 1:**
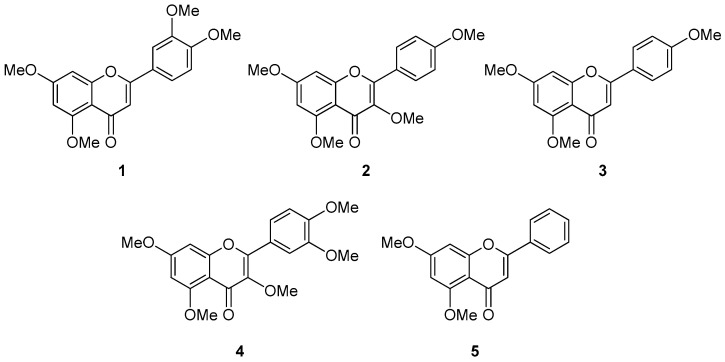
Chemical structures of compounds **1**–**5**.

To discover methoxyflavones with protective properties against skin damage, a phytochemical analysis was conducted on the methanol (MeOH) extract of *Kaempferia parviflora* rhizomes. The crude extract (9.2 g) was subjected to sequential solvent partitioning using *n*-hexane, dichloromethane (CH_2_Cl_2_), ethyl acetate (EtOAc), and *n*-butanol (*n*-BuOH), resulting in four distinct fractions. Among these, the CH_2_Cl_2_-soluble fraction (3.2 g) was found to be particularly rich in methoxyflavones—known as the principal bioactive constituents of *K. parviflora*—as supported by UV absorption profiles and mass spectrometric data from LC/MS, in alignment with previously published reports [39]. Subsequent purification of the CH_2_Cl_2_ fraction was guided by LC/MS and involved multiple chromatographic techniques, including silica gel, reversed-phase C18, and Sephadex LH-20 column chromatography, followed by preparative HPLC. Through this comprehensive isolation process, five distinct methoxyflavones (compounds **1**–**5**) were successfully obtained. The isolated methoxyflavones were identified as 5,7,3′,4′-tetramethoxyflavone (**1**), 3,5,7,4′-tetramethoxyflavone (**2**), 5,7,4′-trimethoxyflavone (**3**), 3,5,7,3′,4′-pentamethoxyflavone (**4**), and 5,7-dimethoxyflavone (**5**) (Figure 1). Structural identification was accomplished by comparing their ^1^H NMR spectral data with previously published values and confirming molecular characteristics through LC/MS analysis [39].

### 3.2. Effects of Methoxyflavones 1–5 on NHDF Viability

**Figure 2:**
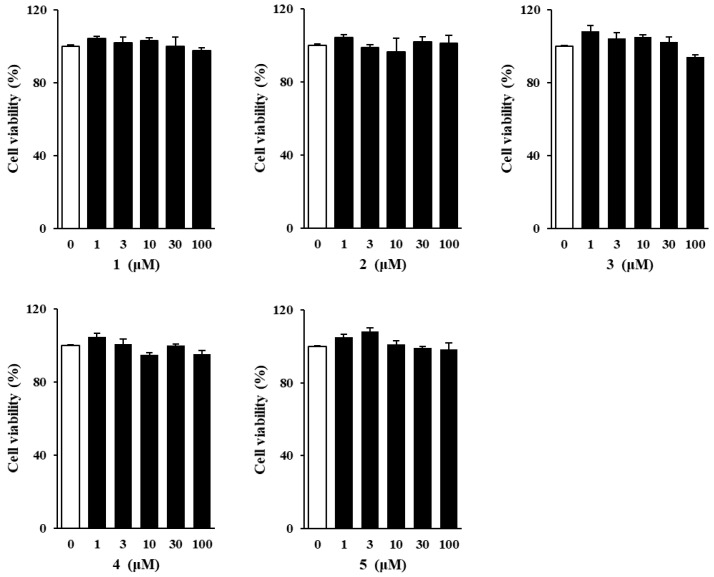
Effect of methoxyflavones **1**–**5** on the viability of normal human dermal fibroblasts (NHDFs). NHDFs were seeded at a density of 1 × 10^4^ cells per well in a 96-well culture plate and cultured for 24 h. Subsequently, the cells underwent serum starvation for an additional 24 h in fresh, serum-free medium. Thereafter, the cells were treated with compounds **1**–**5** at predetermined concentrations and incubated for another 24 h. Cell viability was assessed using the Ez-Cytox kit. Data are presented as mean ± standard error of the mean (SEM) from three independent.

Prior to investigating the protective effects of the isolated methoxyflavones against TNF-α-induced cellular damage, their potential cytotoxicity toward NHDFs was evaluated. Cell viability assays revealed no significant cytotoxic effects at any of the tested concentrations (Figure 2). Based on these findings, subsequent experiments were carried out using a concentration range of 1 to 100 μM.

### 3.3. Effects of Methoxyflavones 1–5 on Intracellular ROS Generation in TNF-α-Stimulated NHDFs

**Figure 3:**
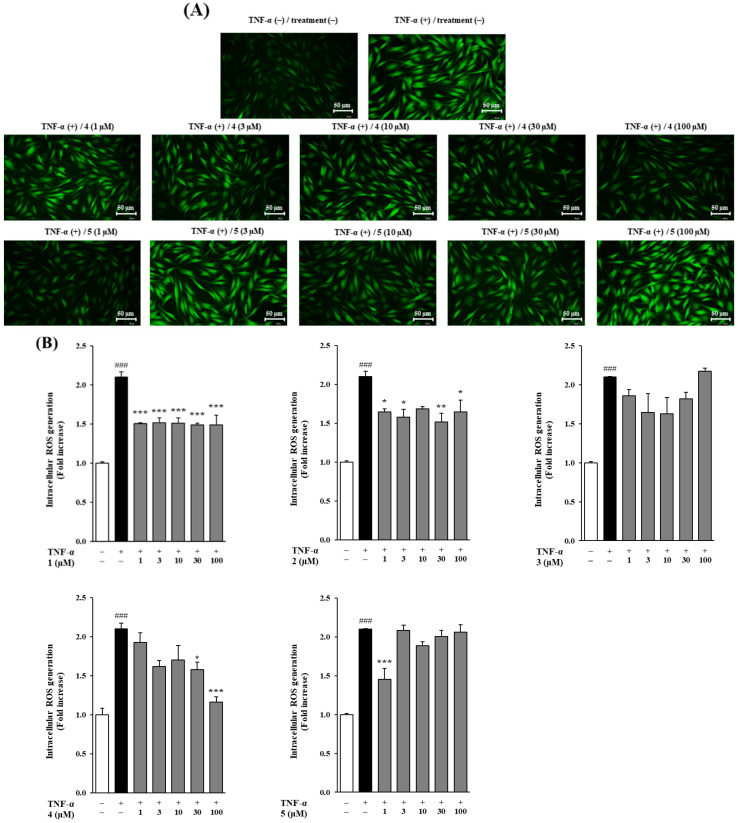
Effects of methoxyflavones **1**–**5** on intracellular reactive oxygen species (ROS) generation in TNF-α-stimulated normal human dermal fibroblasts (NHDFs). NHDFs were seeded at a density of 1 × 10^4^ cells per well in a 96-well black plate and cultured for 24 h. Following a 24 h serum starvation, cells were treated with methoxyflavones **1**–**5** at predetermined concentrations for 1 h, and then exposed to TNF-α (20 ng/mL) and DCFDA (10 μM) for 15 min. After treatment, the DCFDA-stained cells were observed using a fluorescence microscope (scale bar = 50 μm). ROS generation was observed through fluorescence microscope images (**A**) and quantified using a microplate reader (**B**). Data are presented as the mean ± SEM of duplicate experiments. ^###^ *p* < 0.001 versus the control group; * *p* < 0.05, ** *p* < 0.01, and *** *p* < 0.001 versus the TNF-α-treated group.

To evaluate the effects of the isolated methoxyflavones on intracellular ROS production, ROS levels were measured in NHDFs following TNF-α stimulation at 20 ng/mL. TNF-α treatment induced a several-fold increase in ROS production relative to the control group. Treatment with compounds **1**, **2**, **4**, and **5** significantly inhibited TNF-α-induced ROS generation, with ROS levels reduced to approximately 2.10 ± 0.07-fold of control for compounds **1**, **2**, and **4** (*p* < 0.001), and 2.10 ± 0.00-fold for compound **5** (*p* < 0.001). In contrast, compound **3** did not exhibit a significant inhibitory effect on ROS production. Notably, compound **4** further suppressed ROS levels to 1.58 ± 0.09 (*p* < 0.01) and 1.16 ± 0.06 (*p* < 0.001) at concentrations of 30 and 100 μM, respectively, while compound **5** achieved significant inhibition at 1 μM, reducing ROS production to 1.45 ± 0.14 (*p* < 0.001) (Figure 3). These findings indicated that, except for compound **3**, the isolated methoxyflavones effectively inhibited intracellular ROS production, with compound **4** showing superior efficacy at higher concentrations and compound **5** being highly effective at lower concentrations (Figure 3).

### 3.4. Effects of Methoxyflavones 1–5 on MMP-1 and COLIA1 Protein Secretion in TNF-α-Treated NHDFs

**Figure 4:**
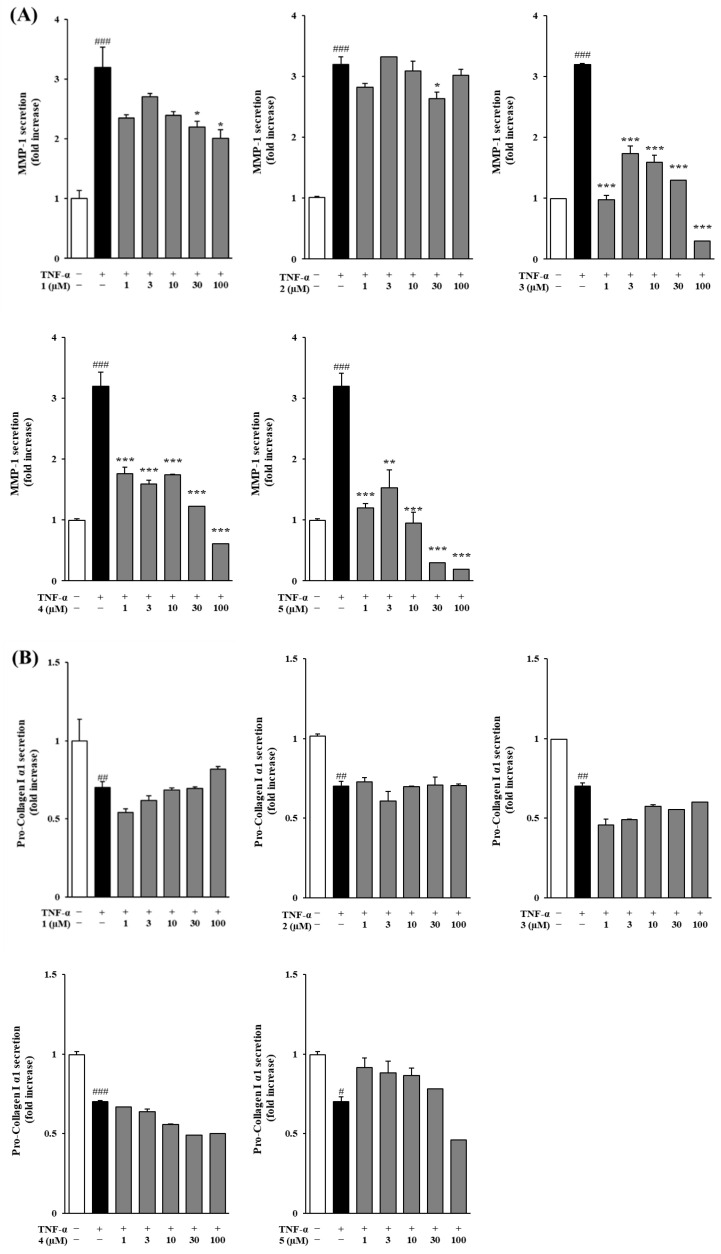
Effect of methoxyflavones **1**–**5** on protein secretion in TNF-α-stimulated normal human dermal fibroblasts (NHDFs). NHDFs were treated with 1, 3, 10, 30, and 100 µM of methoxyflavones **1**–**5** for 1 h, followed by treatment with 20 ng/mL TNF-α for 24 h. MMP-1 (**A**) and pro-collagen I α1 (**B**) levels were measured using ELISA kits. Data are shown as mean ± SEM (n = 3). ^#^ *p* < 0.05, ^##^ *p* < 0.01, and ^###^ *p* < 0.001 versus the control group; * *p* < 0.05, ** *p* < 0.01, and *** *p* < 0.001 versus the TNF-α-treated group.

Matrix metalloproteinases (MMPs), particularly MMP-1 and MMP-9, play a key role in extracellular matrix (ECM) degradation and are closely associated with the progression of skin aging. To investigate the impact of methoxyflavones on ECM integrity, we measured their effects on MMP-1 production and collagen secretion using ELISA (Figure 4). Stimulation with TNF-α (20 ng/mL) led to a marked elevation in MMP-1 levels compared to the untreated control group. However, treatment with methoxyflavones **1**–**5** significantly inhibited MMP-1 secretion compared to the TNF-α group, with fold changes of 3.19 ± 0.34 for compound **1**, 3.19 ± 0.13 for compound **2**, 3.19 ± 0.07 for compound **3**, 3.19 ± 0.23 for compound **4**, and 3.19 ± 0.22 for compound **5** (*p* < 0.001 for all). Notably, treatment with compound **4** at concentrations of 1, 3, 10, 30, and 100 μM further reduced MMP-1 secretion to 1.76 ± 0.11 (*p* < 0.001), 1.59 ± 0.06 (*p* < 0.001), 1.74 ± 0.01 (*p* < 0.001), 1.23 ± 0.09 (*p* < 0.001), and 0.61 ± 0.10 ng/mL (*p* < 0.001), respectively. Similarly, compound **5** decreased MMP-1 secretion to 1.20 ± 0.07 (*p* < 0.001), 1.53 ± 0.30 (*p* < 0.01), 0.95 ± 0.17 (*p* < 0.001), 0.30 ± 0.01 (*p* < 0.001), and 0.19 ± 0.01 ng/mL (*p* < 0.001) at 1, 3, 10, 30, and 100 μM, respectively. Furthermore, TNF-α treatment significantly reduced the secretion of COLIA1. In contrast, treatment with compound **5** promoted COLIA1 secretion compared to the TNF-α group, with a fold change of 0.70 ± 0.03 (*p* < 0.05). Additionally, at concentrations of 1, 3, 10, and 30 μM, compound **5** increased COLIA1 secretion to 0.91 ± 0.06, 0.88 ± 0.07, 0.86 ± 0.05, and 0.78 ± 0.04 ng/mL, respectively.

### 3.5. Spider Chart to Compare the Efficacy of Methoxyflavones 1–5

**Figure 5:**
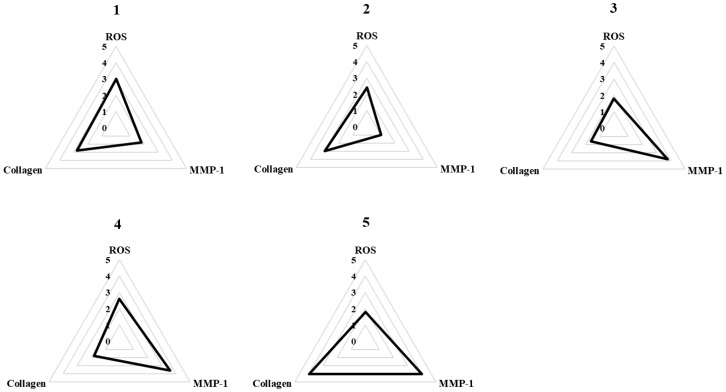
Spider chart for comparing the efficacy of methoxyflavones **1**–**5**. To comprehensively compare the activity of methoxyflavones **1**–**5**, we generated a spider chart that integrates their combined effects on three key parameters: inhibition of ROS production, reduction in MMP-1 secretion, and activation of pro-collagen I α1 expression. For each parameter, the range of measured values was divided into five segments based on the observed maximum and minimum values. Each concentration level was then assigned a rating corresponding to its performance, and the average rating across concentrations was plotted on the chart. In this spider chart, the area enclosed by the plotted values reflects the relative potency of each compound—larger areas indicate greater efficacy.

To compare the biological activities of methoxyflavones **1**–**5**, we assessed their effects across three critical parameters: suppression of ROS generation, inhibition of MMP-1 secretion, and enhancement of collagen production. Each parameter was categorized into five levels based on experimental values, and scores were assigned accordingly to each compound. As illustrated in the spider plot (Figure 5), this comparative analysis revealed that compounds **4** and **5** exhibited the most pronounced effects, particularly in regulating MMP-1 and collagen levels. The larger areas enclosed by these two compounds in the chart highlight their superior performance in modulating these ECM-related markers. These findings indicate that compounds **4** and **5** possess strong potential to mitigate skin aging by maintaining extracellular matrix homeostasis.

### 3.6. Effects of Methoxyflavones 4 and 5 on MAPK Phosphorylation in TNF-α-Treated NHDFs

TNF-α-induced reactive oxygen species (ROS) activate the MAPK signaling pathway through phosphorylation, thereby enhancing the expression of collagen-degrading enzymes and pro-inflammatory cytokines. To elucidate the mechanism by which compounds **4** and **5** inhibit skin aging, we performed Western Blot analysis to evaluate MAPK phosphorylation in TNF-α-treated NHDFs.

**Figure 6:**
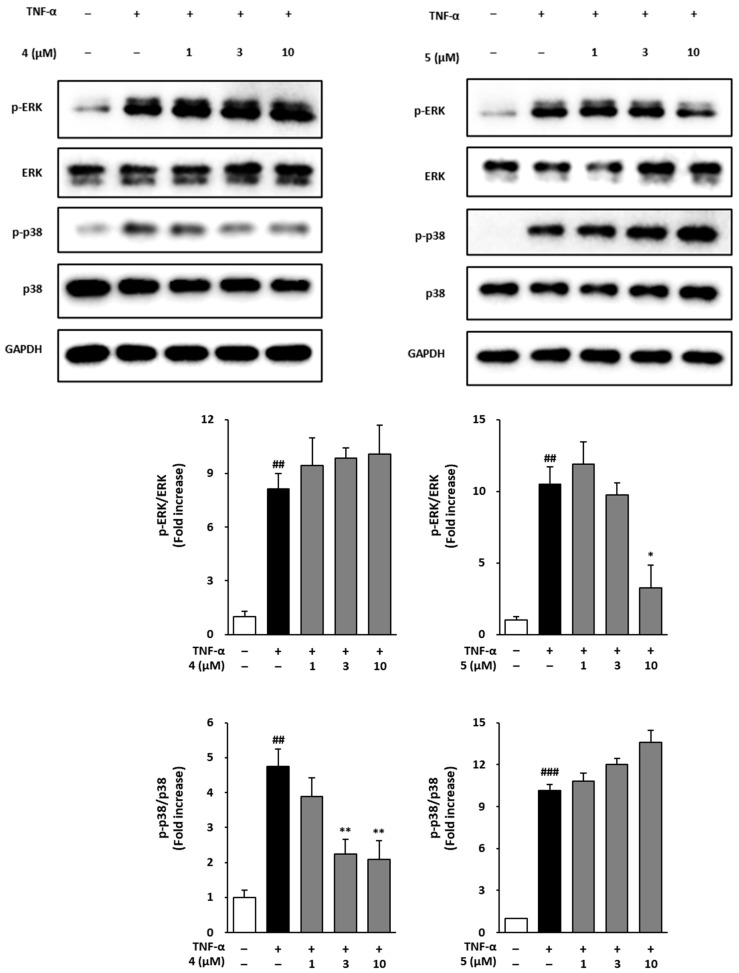
Effect of compounds **4** and **5** on TNF-α-induced MAPK phosphorylation in normal human dermal fibroblasts (NHDFs). Cells were pre-treated with compounds **4** and **5** at concentrations of 1, 3, and 10 µM for 1 h before being exposed to 20 ng/mL TNF-α for 15 min. Immunoblotting was performed to analyze the levels of phosphorylated and total forms of ERK, and p38, with GAPDH serving as a loading control. The results are expressed as fold changes in phosphorylation relative to the control group. Data are presented as mean ± SEM (n = 3). ^##^ *p* < 0.01, and ^###^ *p* < 0.001 versus the control group; * *p* < 0.05, and ** *p* < 0.01 versus the TNF-α-treated group.

As shown in Figure 6, TNF-α exposure markedly increased MAPK phosphorylation. In cells treated with compound **4**, ERK phosphorylation increased by 8.14 ± 0.84-fold compared with the control (*p* < 0.01), and treatment with 1, 3, and 10 µM resulted in 9.44 ± 1.52-, 9.87 ± 0.54-, and 10.06 ± 1.61-fold changes, respectively, without statistical significance. In cells treated with compound **5**, ERK phosphorylation increased by 10.51 ± 1.19-fold (*p* < 0.01), while treatment with 1, 3, and 10 µM led to 11.91 ± 1.54-, 9.75 ± 0.84-, and 3.25 ± 1.57- (*p* < 0.05) fold changes, respectively. Regarding p38 phosphorylation, treatment with compound **4** increased phosphorylation by 4.75 ± 0.49-fold compared with the control (*p* < 0.01), and treatment with 1, 3, and 10 µM resulted in 3.89 ± 0.54-, 2.24 ± 0.42- (*p* < 0.01), and 2.08 ± 0.54- (*p* < 0.01) fold changes, respectively. In cells treated with compound **5**, p38 phosphorylation increased by 10.15 ± 0.42-fold (*p* < 0.001), and treatment with 1, 3, and 10 µM resulted in 10.82 ± 0.55-, 12.00 ± 0.44-, and 13.59 ± 0.08-fold increases, respectively, without statistical significance. These results suggest that compounds **4** and **5** may suppress skin-aging-related protein expression by selectively regulating ERK and p38 phosphorylation within the TNF-α-induced MAPK signaling pathway.

## 4. Discussion

Skin aging arises from a complex interplay of intrinsic and extrinsic factors, with ultraviolet (UV) radiation-induced photoaging recognized as a major contributor to premature cutaneous aging. Upon penetrating the skin, UV light triggers the generation of reactive oxygen species (ROS), leading to oxidative stress and initiating inflammatory cascades [40,41,42,43]. This oxidative stress activates matrix metalloproteinases (MMPs), particularly MMP-1, which specifically targets and degrades type I collagen—one of the key structural proteins in the extracellular matrix (ECM) [44,45]. The breakdown of ECM integrity results in reduced skin firmness and the development of wrinkles, thereby accelerating visible signs of aging. In response to increasing consumer demand for natural and non-toxic anti-aging solutions, significant research efforts have focused on identifying plant-derived compounds with antioxidant, anti-inflammatory, and skin-protective properties [20,46].

*Kaempferia parviflora*, a medicinal plant widely used in Southeast Asia, is well known for its polymethoxyflavones, which exhibit diverse biological activities, including antioxidant and anti-aging effects. Our previous study primarily focused on the quantification and biological evaluation of three major polymethoxyflavones from *K. parviflora* such as 5,7-dimethoxyflavone, 5,7,4′-trimethoxyflavone, and 3,5,7,3′,4′-pentamethoxyflavone, using commercially available compounds [36]. However, the biological mechanisms of less-studied methoxyflavones directly isolated from *K. parviflora* remain largely unexplored.

To address this knowledge gap, the present study involved the isolation and structural elucidation of five methoxyflavones (**1**–**5**) directly from *K. parviflora*, including two additional compounds (**1** and **2**) that were not reported in the previous work. A comprehensive structural characterization was performed using multiple analytical techniques (HPLC–UV chromatograms, UV absorption spectra, MS, and NMR), and all corresponding raw data are provided in the Supplementary Information. Interestingly, the bioactivity and cytotoxicity profiles of the isolated compounds differed from those of the commercial standards, necessitating the use of distinct concentration ranges for biological assays. Under TNF-α-induced oxidative stress conditions, all five compounds were comparatively evaluated, and two representative compounds exhibiting potent anti-aging and protective effects were selected for further mechanistic investigations. Overall, this study provides new insights into the structure–activity relationships (SARs) and distinct molecular mechanisms of *K. parviflora* methoxyflavones in protecting normal human dermal fibroblasts (NHDFs) from oxidative stress and extracellular matrix (ECM) degradation.

Cytotoxicity evaluation confirmed that all five methoxyflavones were non-toxic at concentrations up to 100 μM, thereby supporting their use in subsequent bioactivity assays. ROS assays demonstrated that compounds **1**, **2**, **4**, and **5** significantly reduced intracellular ROS levels in TNF-α-stimulated NHDFs, with compounds **4** and **5** exhibiting dose-dependent inhibition. These potent antioxidant effects suggest that these compounds can protect dermal fibroblasts from oxidative damage, helping to preserve cellular homeostasis and delay inflammation-associated aging responses [47]. Compound **3** did not significantly inhibit ROS generation but effectively suppressed MMP-1 expression, suggesting that it may regulate MMP-1 via an ROS-independent mechanism. This regulation could involve direct interaction with the catalytic site of the MMP-1 enzyme, leading to inhibition of its activity, or modulation of upstream signaling pathways, such as MAPK, NF-κB, or TGF-β, which are known to downregulate MMP-1 expression independently of ROS levels. These observations highlight the possibility that compound **3** exerts anti-aging effects through a distinct ROS-independent mechanism, which warrants further investigation.

Subsequent analysis of extracellular matrix (ECM) regulation demonstrated that all five methoxyflavones markedly inhibited MMP-1 secretion in TNF-α-stimulated NHDFs. Among them, compound **5** significantly restored Pro-Collagen I α1 (COLIA1) protein expression. Compounds **4** and **5** exhibited the strongest effects on these ECM-associated biomarkers, suggesting their capacity to preserve ECM architecture and promote collagen production under inflammatory conditions. These results highlight compounds **4** and **5** as potential therapeutic agents for maintaining ECM homeostasis in inflammation-driven skin aging [48,49].

To facilitate a comparative evaluation of the bioactivities of methoxyflavones **1**–**5**, a radar plot analysis was conducted, integrating three key indicators: ROS inhibition, MMP-1 suppression, and COLIA1 enhancement. The visualized results clearly demonstrate the superior efficacy of compounds **4** and **5**, supporting their potential as lead candidates for skin-protective applications. Based on these findings, compounds **4** and **5**, which exhibited the most pronounced anti-aging effects across multiple assays, were selectively investigated to elucidate their underlying mechanisms. This targeted approach focuses on the most biologically relevant compounds, ensuring experimental consistency and mechanistic clarity.

TNF-α is known to activate MAPKs, which subsequently promote MMP-1 expression through the AP-1 and NF-κB transcription factors [50,51,52,53,54]. Our Western Blot analyses revealed that treatment with compound **5** (10 μM) selectively suppressed ERK phosphorylation, whereas compound **4** (10 μM) inhibited p38 phosphorylation. Given the critical roles of ERK and p38 in upregulating MMP-1 expression and contributing to ECM degradation, these findings suggest that the anti-aging effects of methoxyflavones **4** and **5** may be mediated through the selective modulation of MAPK signaling pathways [55].

Furthermore, it is highly beneficial to interpret the observed anti-aging effects within the broader context of the cellular defense system, particularly the NRF2 (Nuclear factor erythroid 2-related factor 2) signaling pathway. NRF2 serves as a master transcriptional regulator that activates antioxidant response element (ARE)-driven genes, such as HO-1 and NQO-1, to neutralize reactive oxygen species (ROS) and maintain redox homeostasis. Considering that ROS acts as a key upstream activator of MMP-1 expression and a major driver of skin aging, the potential of methoxyflavones **4** and **5** to modulate NRF2 signaling could offer a more fundamental and comprehensive explanation for their bioactivity, complementing the observed MAPK inhibition. The MAPK and NRF2 pathways exhibit intricate crosstalk during oxidative and inflammatory stress responses; specifically, ERK signaling can influence NRF2 stability and transcriptional activity, and vice versa. Therefore, the selective ERK suppression observed with compounds **4** and **5** may not only disrupt the AP-1/MMP-1 axis but also indirectly modulate the cellular redox balance through NRF2 activation. This suggests a potential multi-targeted mechanism of action that enhances the therapeutic relevance of these methoxyflavones. Although our current study primarily confirms MAPK modulation, further research is warranted to investigate the direct activation of NRF2 by compounds **4** and **5**. Future studies should assess NRF2 nuclear translocation and the expression of downstream genes (HO-1, NQO-1) to determine whether their anti-aging effects are mediated solely through MAPK inhibition or involve a synergistic activation of endogenous antioxidant defenses via NRF2 signaling. This integrated perspective will help establish a more definitive mechanistic framework for the therapeutic potential of these compounds.

In addition, the structure–activity relationships (SARs) of the isolated methoxyflavones were investigated. The analysis indicated that the methoxy group at the C-3′ position plays a more significant role in enhancing activity than the methoxy group at C-3, as evidenced by the superior ROS inhibition observed with compounds **1** and **2**. Moreover, the total number of methoxy groups was found to be a crucial factor, with compounds containing four methoxy groups (compounds **1** and **2**) consistently exhibiting greater inhibition of ROS generation compared to others. Conversely, for MMP-1 secretion inhibition, lower methoxy substitution appeared to be more favorable. Compound **5** exhibited the strongest inhibitory effect, while increased methoxy substitution generally led to a gradual decrease in activity. Notably, compound **4**, which contains five methoxy groups, showed moderate MMP-1 secretion inhibition. This deviation can be attributed to the enhanced lipophilicity conferred by multiple methoxy groups, which may improve membrane permeability even as higher methoxy substitution trends toward reduced intrinsic activity [56]. According to the previous report, 3,5,7,3′,4′-pentamethoxyflavone demonstrated superior superoxide-scavenging activity compared to its unmethylated analogs and glycosides [57]. Remarkably, this pentamethoxyflavone exhibited 26-fold greater skin permeability than quercetin in a nude mouse model, underscoring its potential for enhanced dermal absorption. These findings collectively suggest that *O*-methoxylation is a promising strategy for improving the topical delivery and bioactivity of flavonoids [57].

**Figure 7:**
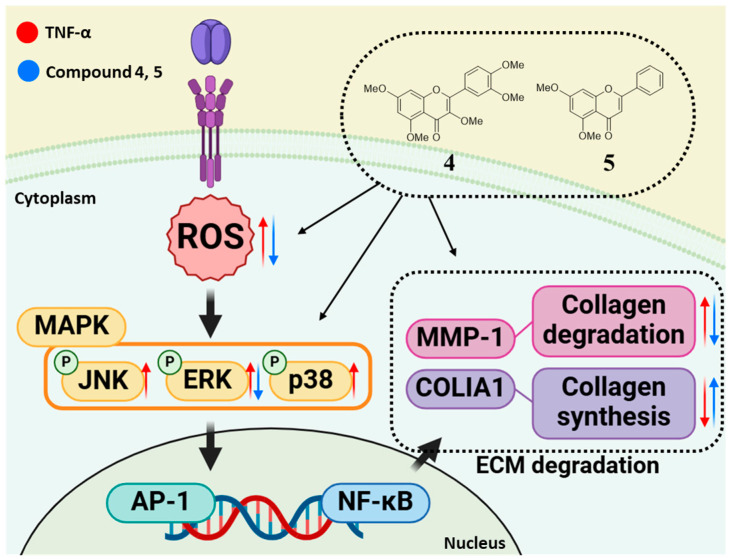
Schematic illustration of the potential protective effect of compounds **4** and **5** in TNF-α-induced NHDFs. Red arrows indicate TNF-α–induced increases or decreases, whereas blue arrows represent the inhibition or reversal of these changes by compounds **4** and **5**.

This study identified methoxyflavones **4** and **5** as effective modulators of TNF-α-induced cellular responses in normal human dermal fibroblasts (NHDFs). These compounds were shown to reduce oxidative stress, inhibit MMP-1 secretion, promote COLIA1 expression, and suppress ERK phosphorylation, highlighting their potential as multifunctional agents for combating skin aging (Figure 7). Nevertheless, as these findings are based solely on in vitro experiments, further in vivo studies are required to evaluate their pharmacokinetic profiles, skin permeability, and overall bioavailability. Clinical validation will also be crucial to determine their safety and efficacy in human populations. Upon confirmation, methoxyflavones **4** and **5** may serve as promising active ingredients in cosmeceutical or dermatological formulations aimed at protecting skin from oxidative and inflammatory damage. This work expands the current understanding of flavonoid-based anti-aging interventions and emphasizes the importance of continued mechanistic and translational research in this area.

## 5. Conclusions

Through LC–MS-guided phytochemical analysis of the methanolic extract of *K*. *parviflora* rhizomes, five methoxyflavones were isolated and identified. Among them, compounds **4** and **5** exhibited the most potent biological activities against TNF-α-induced oxidative stress and extracellular matrix (ECM) degradation in normal human dermal fibroblasts (NHDFs). These two methoxyflavones markedly reduced intracellular reactive oxygen species (ROS), suppressed MMP-1 secretion, and enhanced COLIA1 expression, thereby preserving ECM integrity and preventing collagen breakdown—key processes involved in skin aging. Mechanistically, compound **5** selectively inhibited ERK phosphorylation, while compound **4** suppressed p38 phosphorylation in TNF-α-stimulated NHDFs, suggesting that their anti-aging effects are mediated through distinct yet complementary modulation of MAPK signaling pathways. Collectively, these findings highlight methoxyflavones **4** and **5** as dual-acting antioxidant and ECM-protective agents that exert anti-aging effects via selective regulation of ERK and p38 activation. However, it should be noted that the present study was conducted exclusively under in vitro conditions, which may limit the direct extrapolation of these results to in vivo systems. Therefore, further investigations using animal models and clinical studies are warranted to validate the anti-photoaging efficacy and translational potential of these compounds, and to explore their applicability in cosmeceutical formulations.

## Data Availability

The original contributions presented in this study are included in the article/supplementary material. Further inquiries can be directed to the corresponding authors.

## Supplementary Materials

The following are available online at https://www.mdpi.com/article/10.3390/foods14234012/s1: Figure S1: The UV chromatogram of CH_2_Cl_2_ soluble fraction from the LC/MS analysis (detection wavelength: 254 nm); Figure S2: The total ion chromatogram (TIC) of CH_2_Cl_2_ soluble fraction from the LC/MS analysis (positive-ion mode); Figure S3: (A) The UV chromatogram of LC/MS (detection wavelength: 315 nm) (B) UV and MS spectrum for compound **1**; Figure S4: The ^1^H NMR spectrum of **1** (CD_3_OD, 850 MHz); Figure S5: (A) The UV chromatogram of LC/MS (detection wavelength: 315 nm) (B) UV and MS spectrum for compound **2**; Figure S6: The ^1^H NMR spectrum of **2** (CDCl_3_, 850 MHz); Figure S7: (A) The UV chromatogram of LC/MS (detection wavelength: 315 nm) (B) UV and MS spectrum for compound **3**; Figure S8: The ^1^H NMR spectrum of **3** (CDCl_3_, 850 MHz); Figure S9: (A) The UV chromatogram of LC/MS (detection wavelength: 315 nm) (B) UV and MS spectrum for compound **4**; Figure S10: The ^1^H NMR spectrum of **4** (CDCl_3_, 850 MHz); Figure S11: (A) The UV chromatogram of LC/MS (detection wavelength: 315 nm) (B) UV and MS spectrum for compound **5**; Figure S12: The ^1^H NMR spectrum of **5** (CDCl_3_, 850 MHz); Materials and Methods.
