# Supplementary material for: Anti-Skin Aging Potential of Methoxyflavones from Kaempferia parviflora Against TNF-α-Induced Oxidative Stress and Photoaging in Normal Human Dermal Fibroblasts
Source: Foods. 2025 Nov 23;14(23):4012. doi: 10.3390/foods14234012 (PMC12691992; doi:10.3390/foods14234012)
Supplement: Supplementary file 1 [file foods-14-04012-s001.zip › foods-3922261-supplementary.pdf]

Supplementary Data

Anti-Skin Aging Potential of Methoxyflavones from *Kaempferia parviflora* Against TNF-α-Induced Oxidative Stress and Photoaging in Normal Human Dermal Fibroblasts

Si-young Ahn [1,†], Se Yun Jeong [2,†], Bum Soo Lee [2], Yun Seok Joh [2], Hamed Hamishehkar [3,4], Sullim Lee [1,*] and Ki Hyun Kim [2,*]

[1] Department of Life Science, College of Bio-Nano Technology, Gachon University, Seongnam 13120, Republic of Korea; sy990303@gachon.ac.kr
[2] School of Pharmacy, Sungkyunkwan University, Suwon 16419, Republic of Korea; dlawktkark@naver.com (S.Y.J.); kosboybs@naver.com (B.S.L.); ysjoh05@g.skku.edu (Y.S.J.)
[3] Drug Applied Research Center, Tabriz University of Medical Sciences, Tabriz 51368, Iran; hamishehkarh@tbzmed.ac.ir
[4] Research Center of New Material and Green Chemistry, Khazar University, 41 Mehseti Street, Baku AZ1096, Azerbaijan
* Correspondence: sullimlee@gachon.ac.kr (S.L.); khkim83@skku.edu (K.H.K.); Tel.: +82-31-290-7700 (K.H.K.)
† These authors contributed equally to this study.

Supplementary data Contents:

Figure S1. The UV chromatogram of CH_2Cl_2 soluble fraction from the LC/MS analysis (detection wavelength: 254 nm) ……………………S3
Figure S2. The total ion chromatogram (TIC) of CH_2Cl_2 soluble fraction from the LC/MS analysis (positive-ion mode) ………………………S4
Figure S3. (A) The UV chromatogram of LC/MS (detection wavelength: 315 nm) (B) UV and MS spectrum for compound **1**……………S5
Figure S4. The ^{1}H NMR spectrum of **1** (CD_3OD, 850 MHz) ……………………………………………………………………S6
Figure S5. (A) The UV chromatogram of LC/MS (detection wavelength: 315 nm) (B) UV and MS spectrum for compound **2**……………S7
Figure S6. The ^{1}H NMR spectrum of **2** ($CDCl_3$, 850 MHz) ……………………………………………………………………S8
Figure S7. (A) The UV chromatogram of LC/MS (detection wavelength: 315 nm) (B) UV and MS spectrum for compound **3**……………S9
Figure S8. The ^{1}H NMR spectrum of **3** ($CDCl_3$, 850 MHz) ……………………………………………………………………S10
Figure S9. (A) The UV chromatogram of LC/MS (detection wavelength: 315 nm) (B) UV and MS spectrum for compound **4**……………S11
Figure S10. The ^{1}H NMR spectrum of **4** ($CDCl_3$, 850 MHz) ……………………………………………………………………S12
Figure S11. (A) The UV chromatogram of LC/MS (detection wavelength: 315 nm) (B) UV and MS spectrum for compound **5**…………S13
Figure S12. The ^{1}H NMR spectrum of **5** ($CDCl_3$, 850 MHz) ……………………………………………………………………S14
Materials and Methods……………………………………………………………………………………………………S15

Figure S1. The UV chromatogram of CH_2Cl_2 soluble fraction from the LC/MS analysis (detection wavelength: 254 nm)

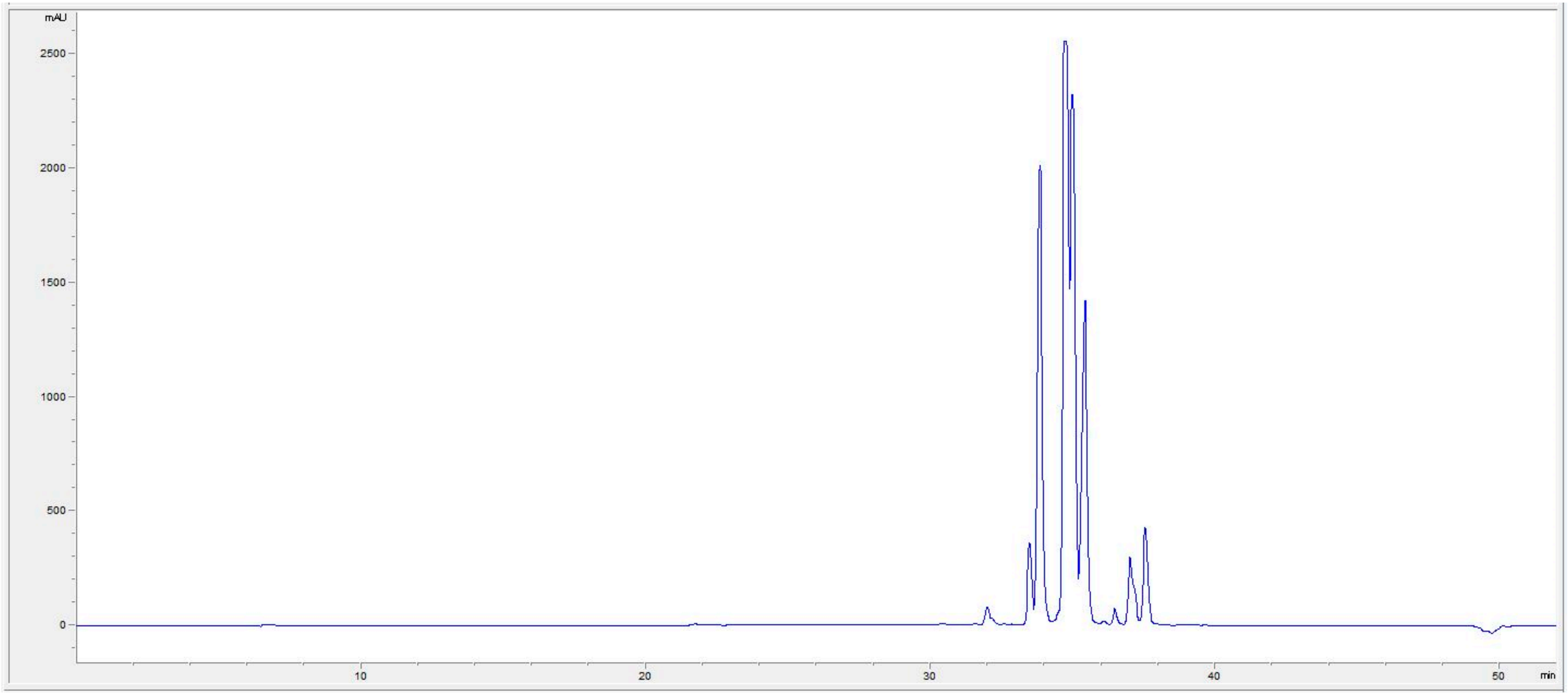

Figure S2. The total ion chromatogram (TIC) of CH_2Cl_2 soluble fraction from the LC/MS analysis (positive-ion mode)

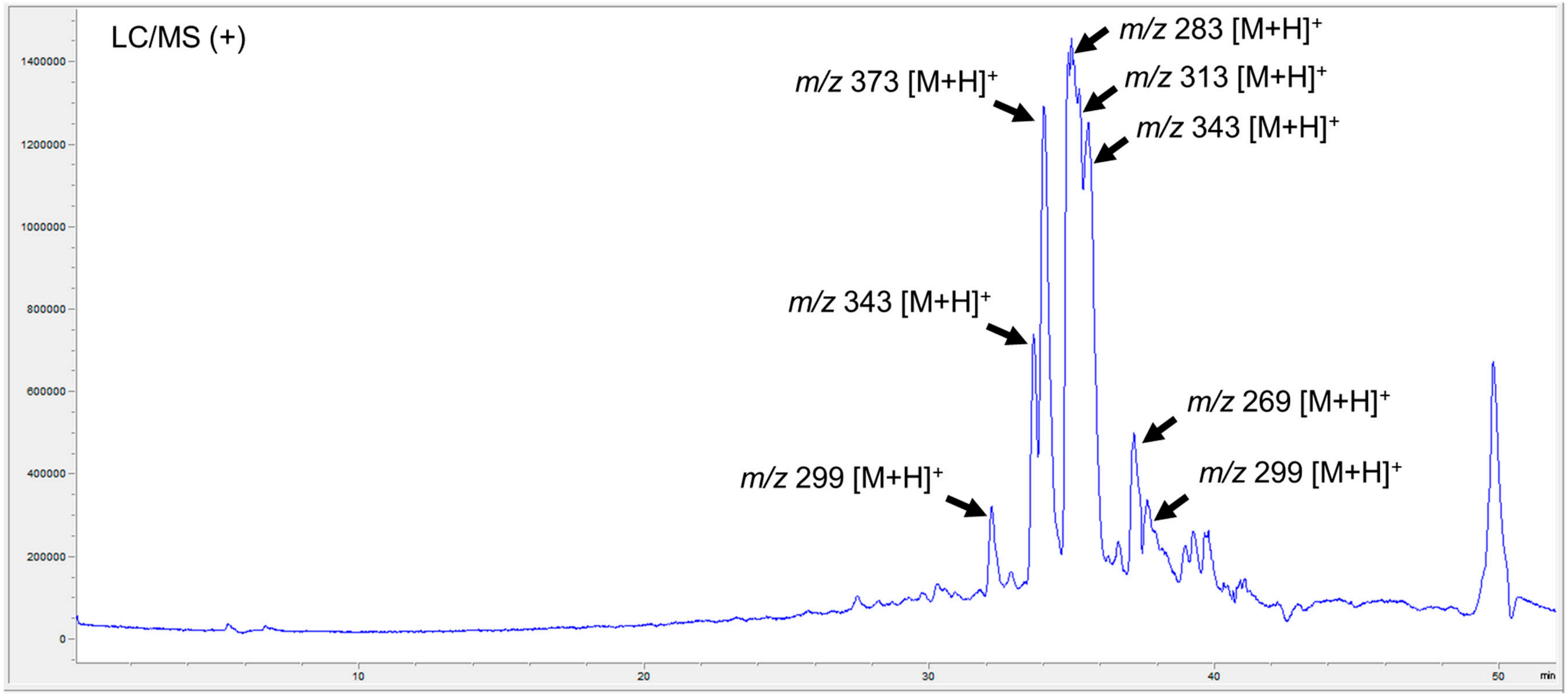

Figure S3. (A) The UV chromatogram of LC/MS (detection wavelength: 315 nm) (B) UV and MS spectrum for compound **1**

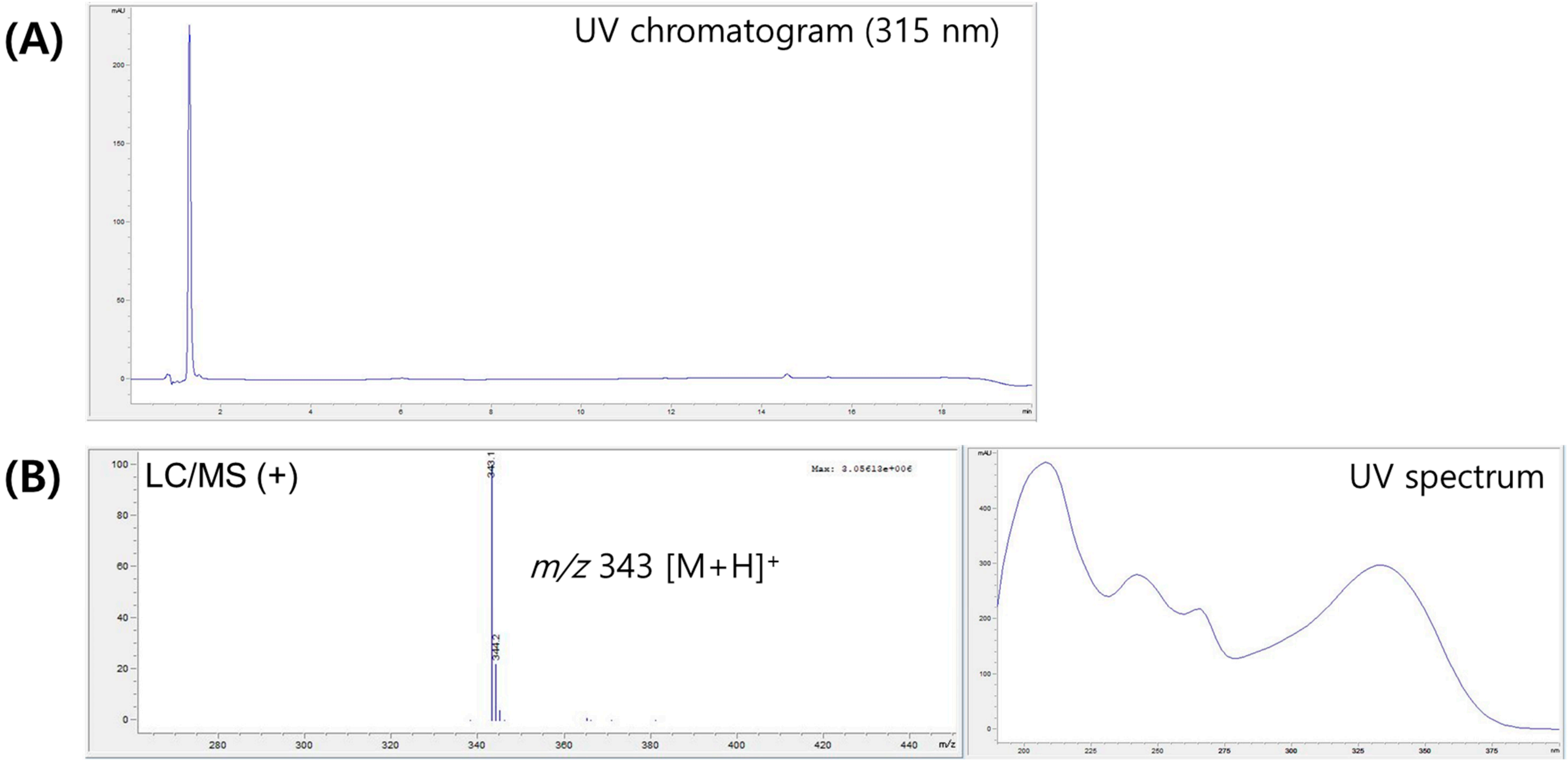

Figure S4. The ^{1}H NMR spectrum of **1** (CD_3OD, 850 MHz)

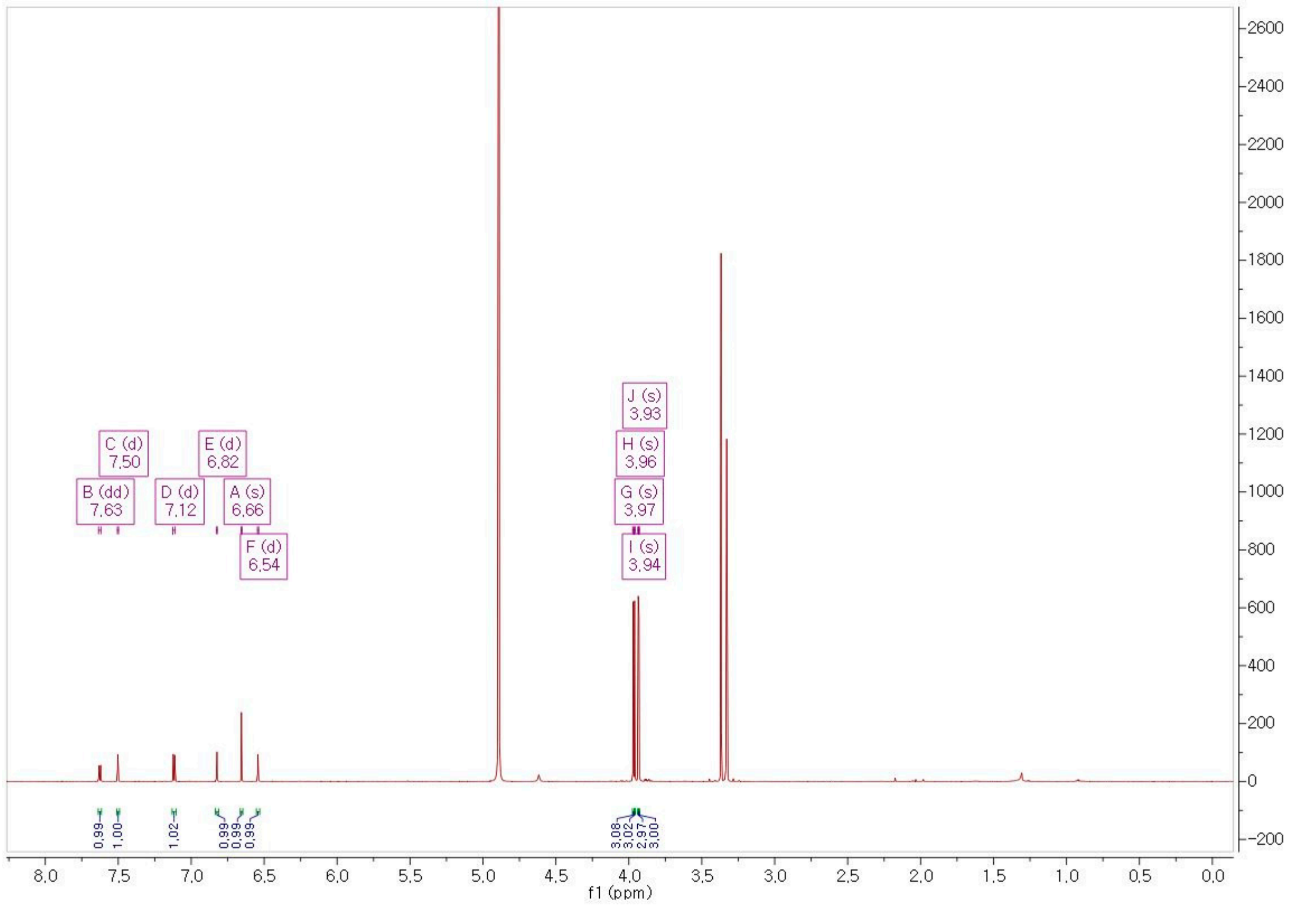

Figure S5. (A) The UV chromatogram of LC/MS (detection wavelength: 315 nm) (B) UV and MS spectrum for compound **2**

(A)

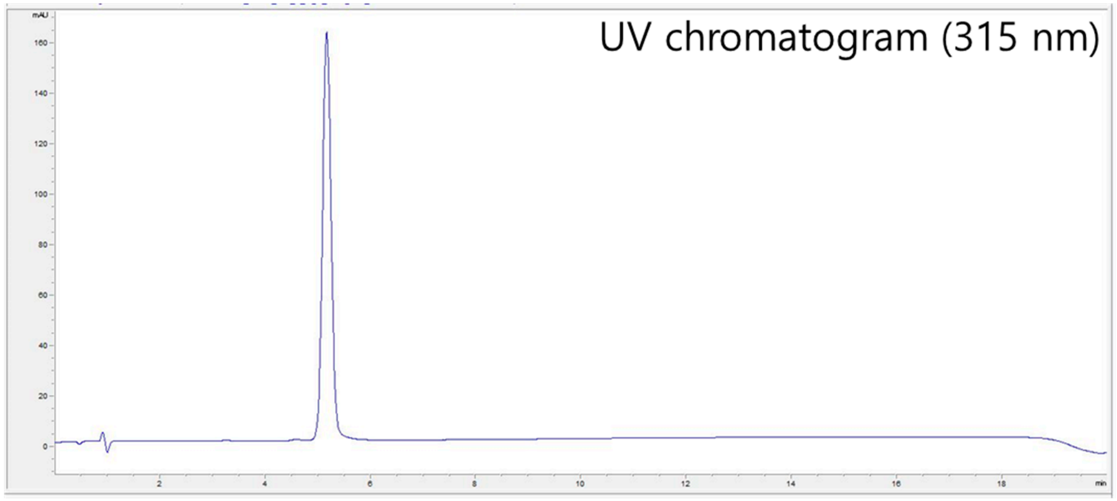

(B)

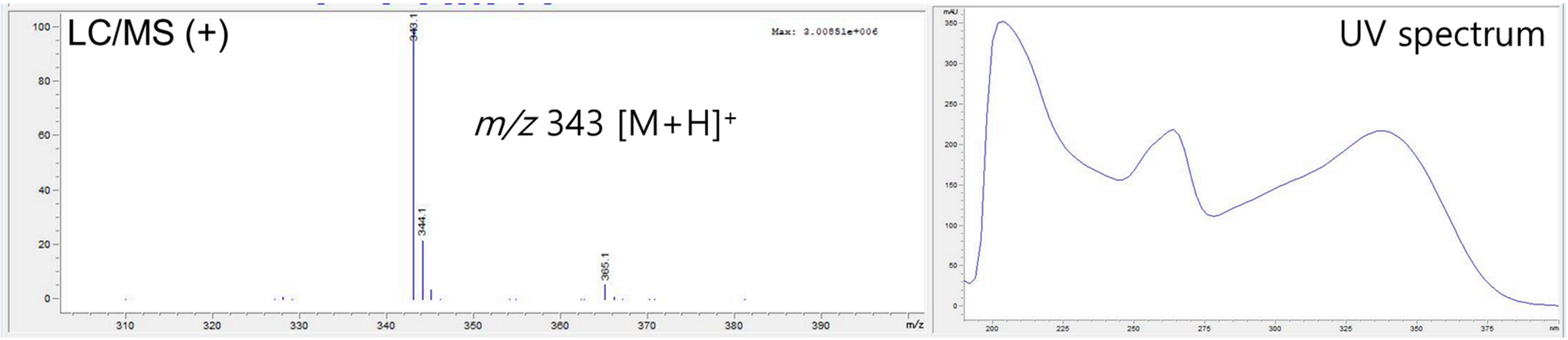

Figure S6. The ^{1}H NMR spectrum of **2** ($CDCl_3$, 850 MHz)

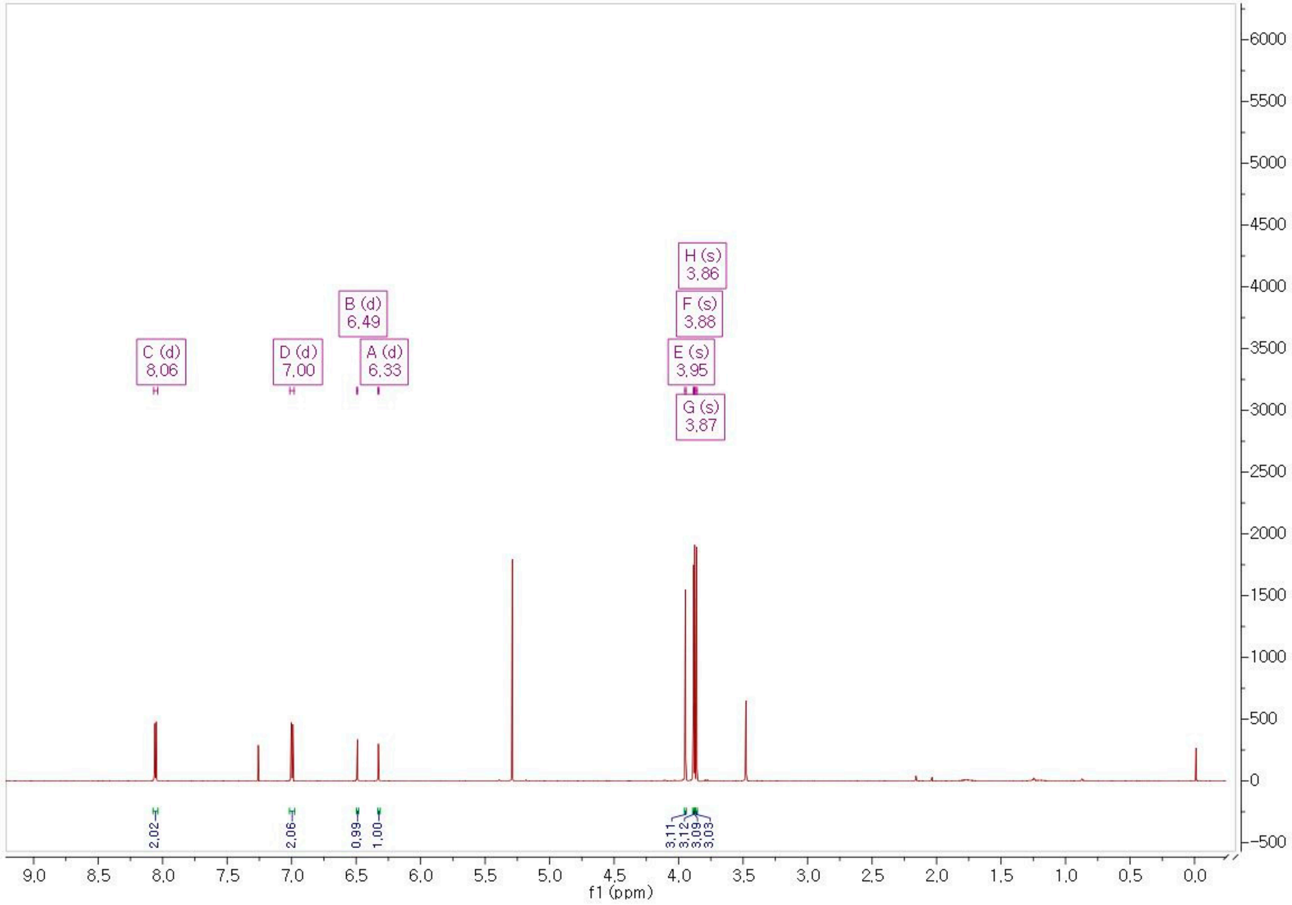

Figure S7. (A) The UV chromatogram of LC/MS (detection wavelength: 315 nm) (B) UV and MS spectrum for compound **3**

(A)

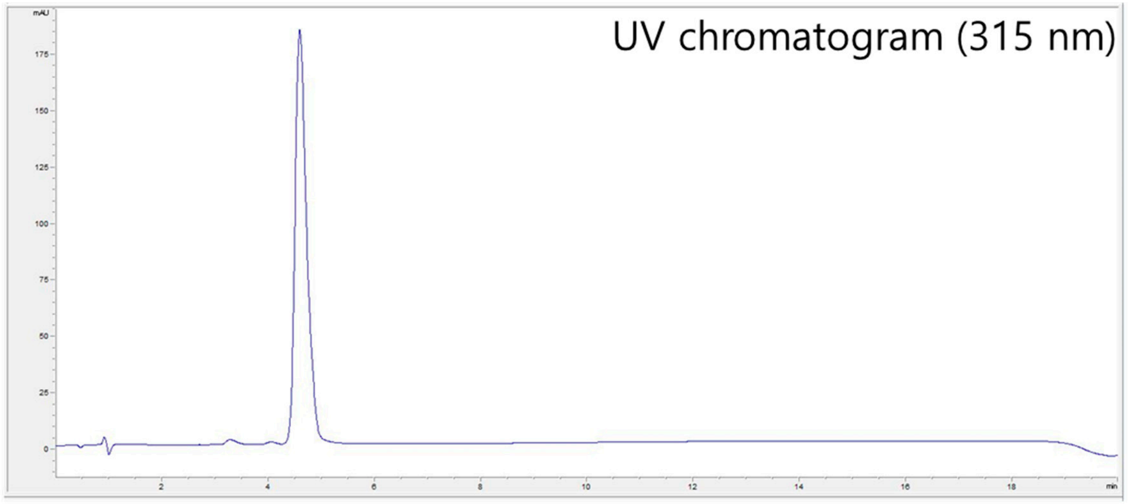

(B)

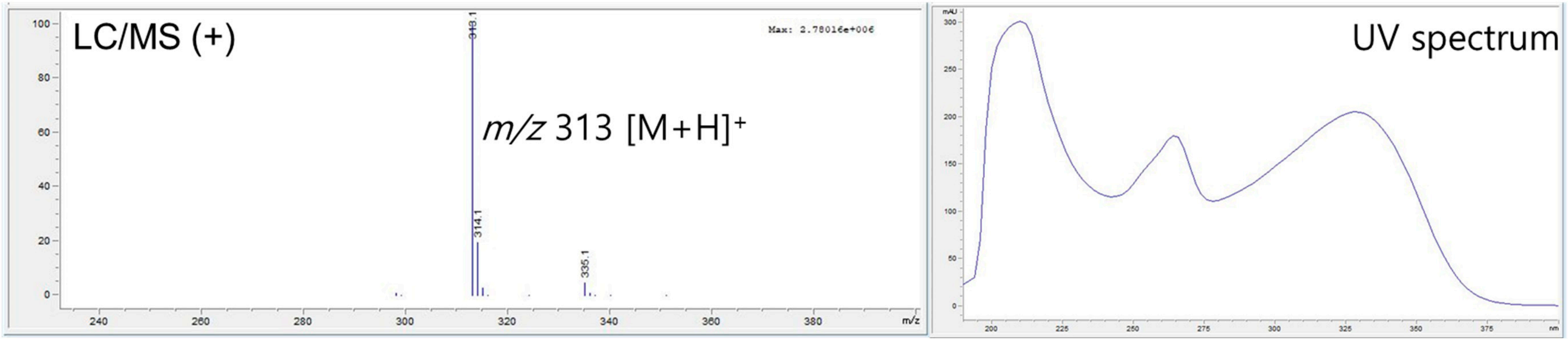

Figure S8. The ^{1}H NMR spectrum of **3** ($CDCl_3$, 850 MHz)

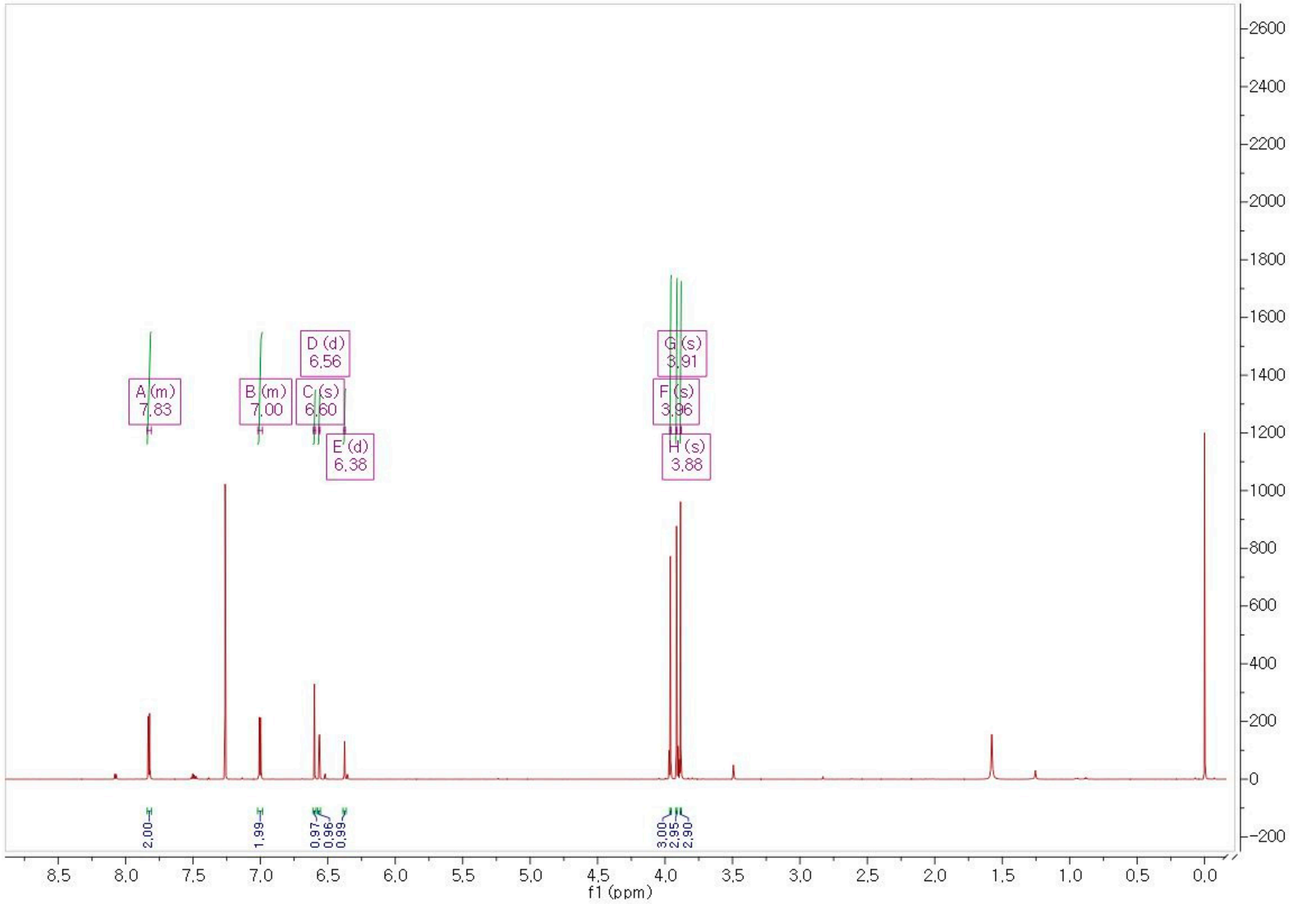

Figure S9. (A) The UV chromatogram of LC/MS (detection wavelength: 315 nm) (B) UV and MS spectrum for compound **4**

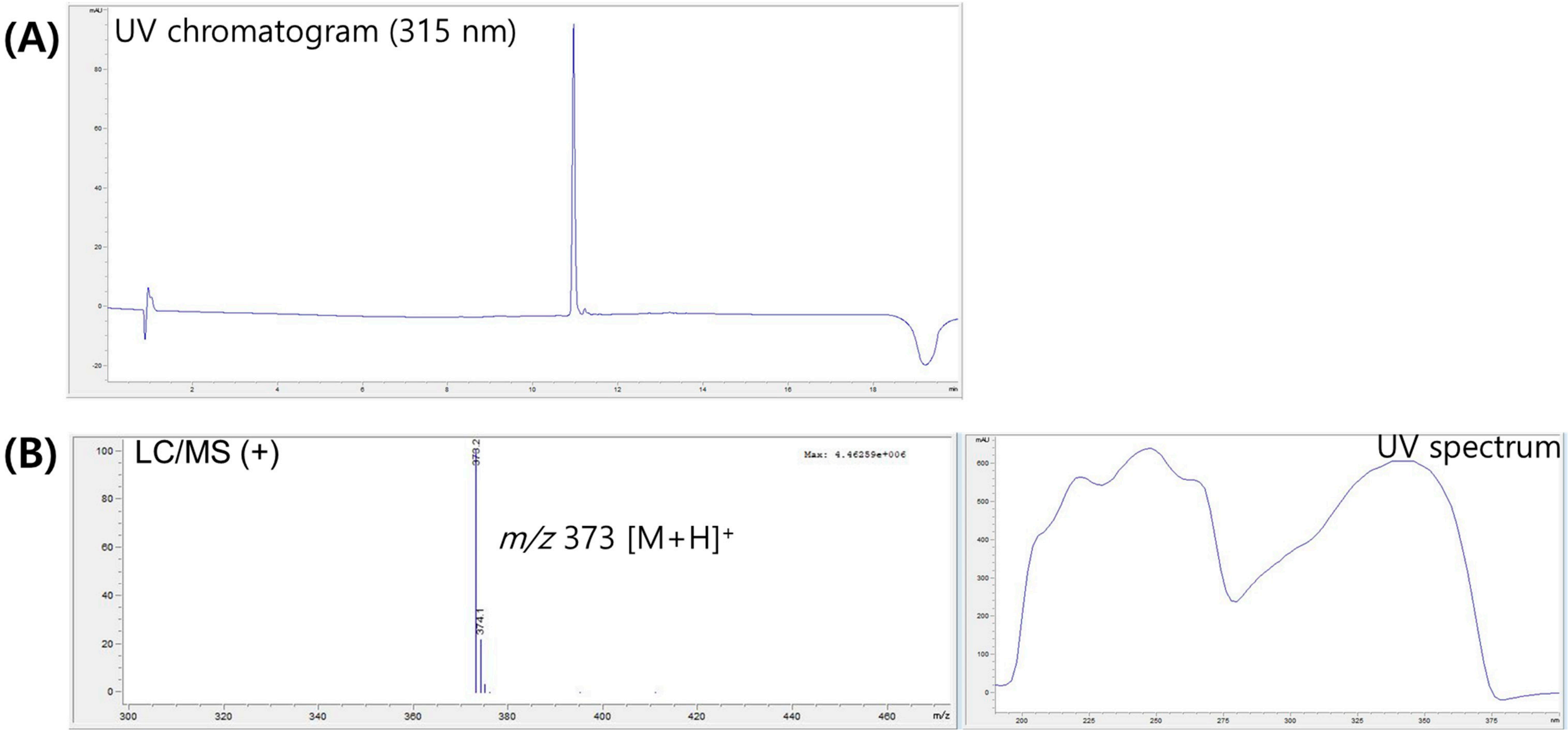

Figure S10. The ^{1}H NMR spectrum of **4** ($CDCl_3$, 850 MHz)

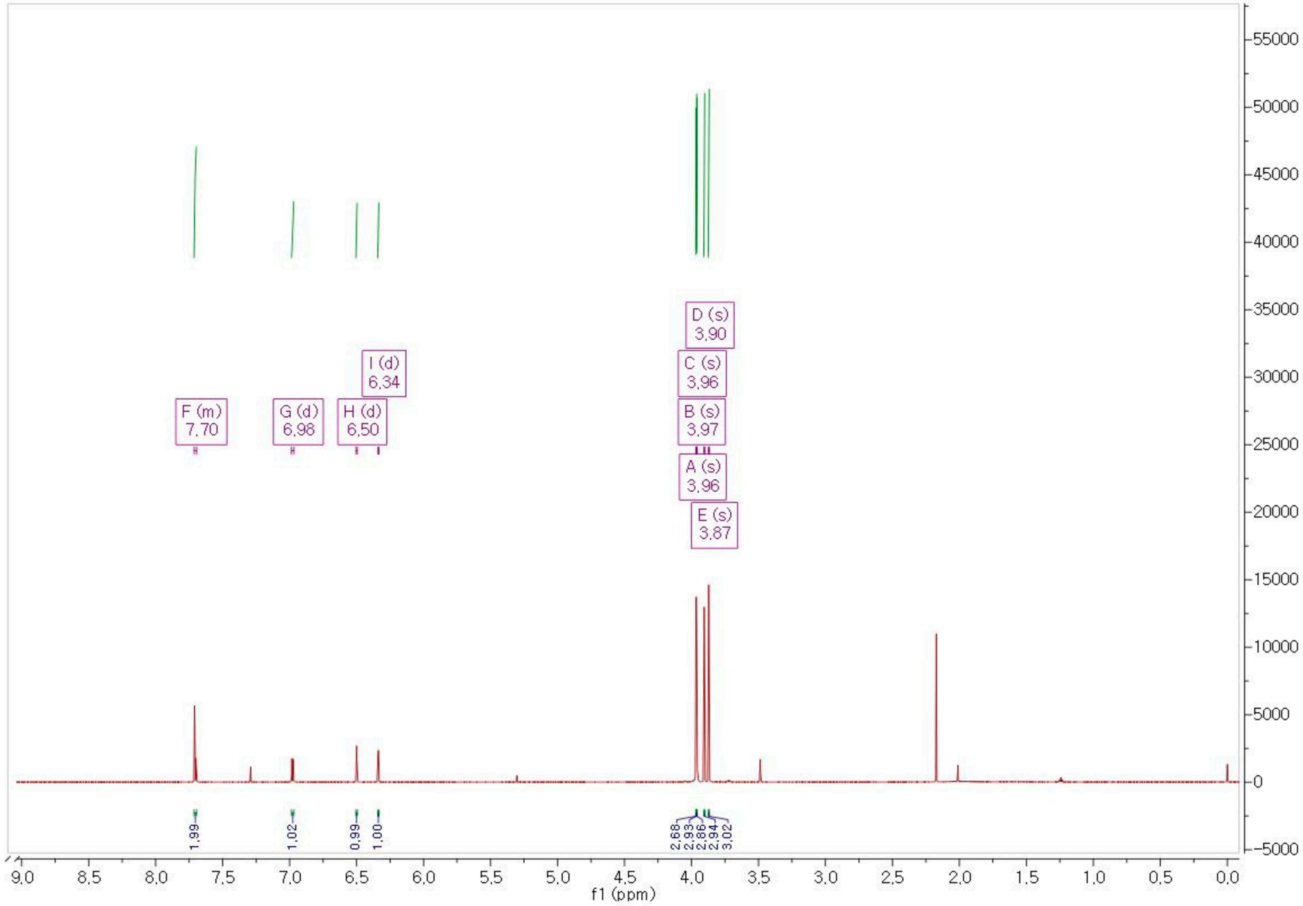

Figure S11. (A) The UV chromatogram of LC/MS (detection wavelength: 315 nm) (B) UV and MS spectrum for compound **5**

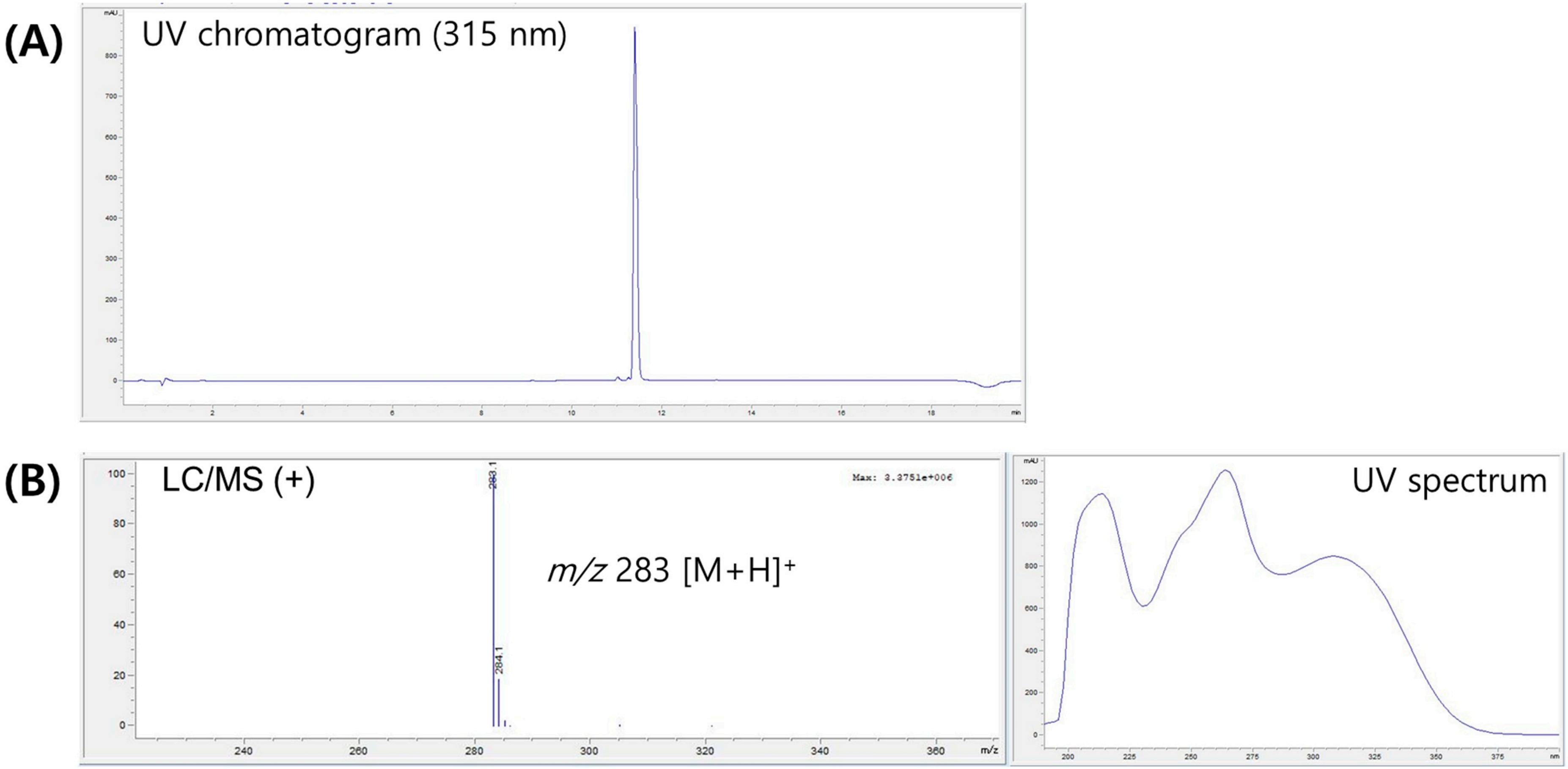

Figure S12. The ^{1}H NMR spectrum of **5** ($CDCl_3$, 850 MHz)

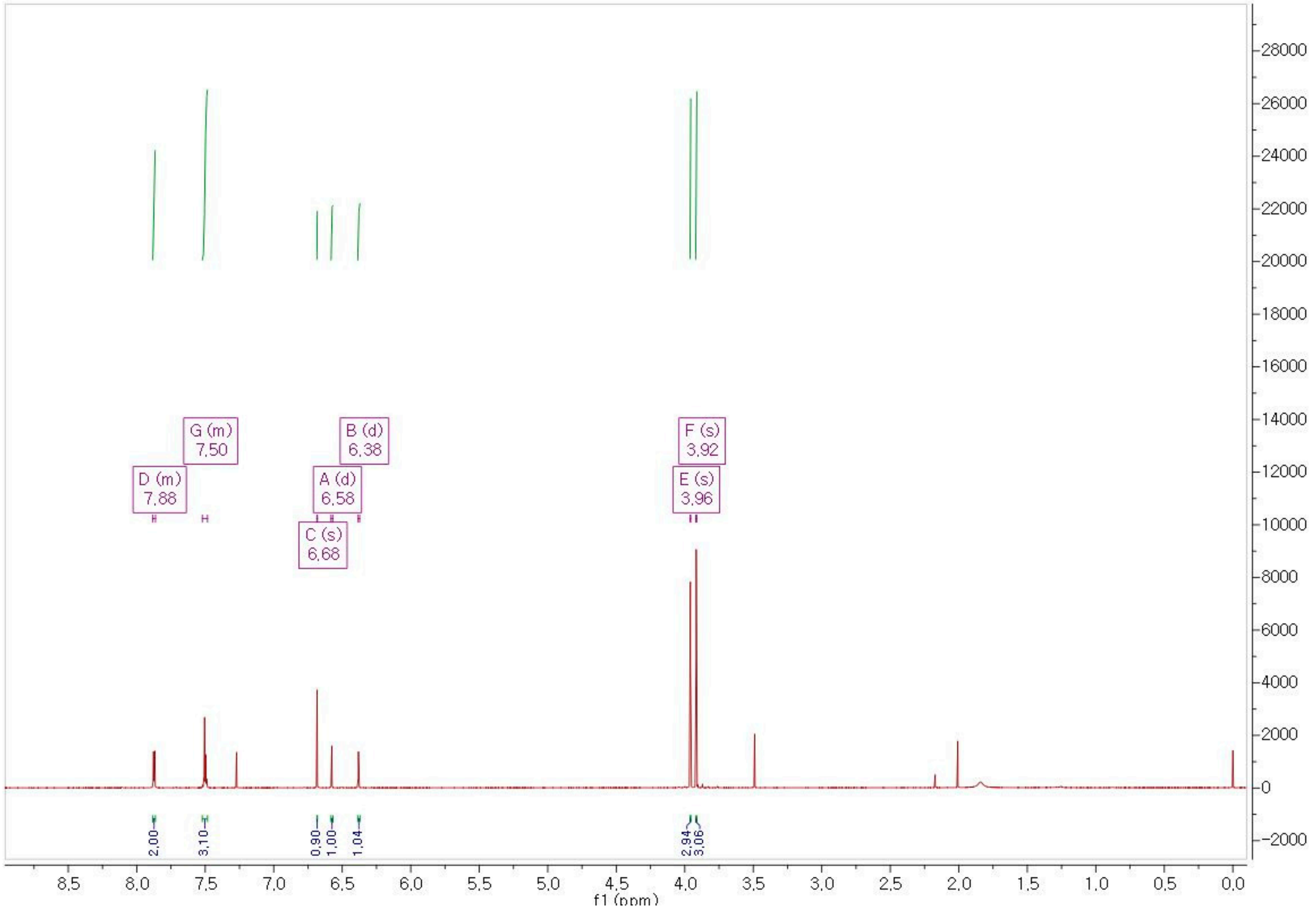

Materials and Methods

Chemicals

All solvents utilized in this research were of analytical grade and sourced from Samchun Chemical Co., Ltd. (Seoul, South Korea). Deuterated solvents for NMR analysis were procured from Cambridge Isotope Laboratories (Tewksbury, MA, USA). For LC–MS analyses, LC–MS grade acetonitrile (MeCN), methanol (MeOH), and formic acid (≥ 98.0% purity) were purchased from Fisher Scientific (Ottawa, ON, Canada).

General experimental procedures

Nuclear magnetic resonance (NMR) spectra were recorded on a Bruker AVANCE III HD 850 MHz spectrometer (Bruker, Karlsruhe, Germany) equipped with a 5 mm TCI CryoProbe, operating at a proton frequency of 850 MHz. Chemical shifts (*δ*) are expressed in parts per million (ppm) and referenced to residual solvent signals: 3.310 ppm for CD_3OD and 7.260 ppm for $CDCl_3$ (Cambridge Isotope Laboratories, Tewksbury, MA, USA). Preparative HPLC separations were conducted using a Waters 1525 Binary Pump system combined with a Waters 996 Photodiode Array Detector (Waters Corporation, Milford, CT, USA), and separation was achieved on a Hector-A C18 column (250 × 21.2 mm, 5 µm particle size; RStech, Chungcheongbuk-do, Korea) at a flow rate of 5 mL/min. For semi-preparative purposes, a Shimadzu Prominence HPLC platform outfitted with SPD-20A/20AV UV–Vis detectors (Shimadzu, Tokyo, Japan) was used in conjunction with a Phenomenex Luna Phenyl-Hexyl column (250 × 10 mm, 10 µm; Phenomenex, Torrance, CA, USA), operated at 2 mL/min. LC–MS analyses were performed using an Agilent 1200 Series HPLC system equipped with a diode array detector and interfaced with a 6130 Series electrospray ionization (ESI) mass spectrometer (Agilent Technologies, Santa Clara, CA, USA). Chromatographic separation was achieved on a Kinetex C18 100 Å column (100 × 2.1 mm, 5 µm; Phenomenex), operated at a flow rate of 0.3 mL/min. Open-column chromatography employed Silica gel 60 (230–400 mesh) and C18-reversed phase silica gel (230–400 mesh) from Merck (Darmstadt, Germany) as stationary phases. Gel filtration chromatography was carried out using Sephadex LH-20 resin (Pharmacia, Uppsala, Sweden). Thin-layer chromatography (TLC) analyses were performed on Merck-

precoated silica gel F254 and RP-C18 F254s plates, with spots visualized under UV illumination or by heating following anisaldehyde–sulfuric acid spray application.

Plant material

Rhizomes of *Kaempferia parviflora* Wall. were collected in January 2020 from Warorot Market, located in Chiang Mai City, Northern Thailand. The plant material was taxonomically authenticated by one of the authors (K. H. Kim), and a corresponding voucher specimen (SKKU-BG 1908) has been preserved in the herbarium of the School of Pharmacy, Sungkyunkwan University, Suwon, Republic of Korea.

Cell Culture and Sample Preparation

Normal human dermal fibroblasts (NHDFs) were sourced from PromoCell GmbH (Heidelberg, Germany). Cells were maintained in Dulbecco's Modified Eagle Medium (DMEM; Corning, Manassas, VA, USA) supplemented with 10% fetal bovine serum (FBS; Atlas, Fort Collins, CO, USA) and 1% penicillin-streptomycin (Gibco, Grand Island, NY, USA). Cultures were incubated at 37°C in a humidified atmosphere containing 5% CO_2. Prior to treatment, all test samples were initially dissolved in dimethyl sulfoxide (DMSO) and then further diluted with DMEM to reach the desired final concentrations for cellular assays.

Cell Viability Measurement

To assess the cytotoxic effects of the isolated compounds on NHDFs, cells were plated in 96-well microplates at a density of 1×10^4 cells per well and incubated for 24 h. To achieve cell cycle synchronization, the medium was then replaced with serum-free DMEM, and the cells were further incubated for another 24 h. Subsequently, the test compounds were applied to the synchronized cells. After 24 h of treatment, Ez-Cytox reagent (10%) was added to each well, and following a 1-h incubation, absorbance was measured at 450 nm using a SPARK 10M microplate

reader (Tecan).
